# Missing species among Mediterranean non-Siphonophoran Hydrozoa

**DOI:** 10.1007/s10531-015-0859-y

**Published:** 2015-01-20

**Authors:** Cinzia Gravili, Stanislao Bevilacqua, Antonio Terlizzi, Ferdinando Boero

**Affiliations:** 1 Laboratory of Zoology and Marine Biology, Dipartimento di Scienze e Tecnologie Biologiche e Ambientali, Di.S.Te.B.A., Università del Salento, Via Prov.le Lecce-Monteroni, 73100 Lecce, Italy; 2 CNR-ISMAR, Via de Marini, 6, 16149 Genoa, Italy

**Keywords:** Biodiversity, Hydrozoa, Extinction, Confidence of extinction index

## Abstract

Hydrozoa of the Mediterranean Sea are well known and a recent monograph covers 457 species. Mediterranean non-Siphonophoran Hydrozoa comprises 398 species, an increasing number due to continuous updates, representing about 10 % of the 3,702 currently valid species reported in a recent world assessment of hydrozoan diversity. Many new records are non indigenous species, previously described species that occurred elsewhere and whose arrival was presumably caused by human activities. However, many species reported in the past are not recorded in recent times. Realistic assessments of species pools require addition of new species, but also subtraction of species not found since a certain period. With the confidence of extinction index, cases of putative extinction can be raised. Out of the 398 known species, only 162 (41 %) have been reported in the last decade, while 53 (13 %) are not recorded in the literature since at least 41 years. According to the confidence of extinction index, 60 % of the 53 missing species are extinct, and 11 % are putatively extinct from the basin. From a biogeographical point of view, the missing species are: 34 % endemic, 19 % boreal, 15 % Mediterranean-Atlantic, 11 % Indo-Pacific, 11 % circumtropical, 4 % cosmopolitan, 2 % tropical-Atlantic, 4 % non-classifiable. Fluctuations in species composition into a certain area cause heavy variability in the expression of both structural and functional biodiversity. As consequence, the regional biodiversity should be analyzed through its temporal evolution, to detect changes and their possible causes. This approach has profound consequences on biodiversity assessments and also on the compilation of red lists.

## Introduction

The question “How many species are there in the oceans?” provides the key to discover what we know and what we do not know about the life in the seas (Mora et al. 2011). Conservation biologists try to identify the areas in the world where effective conservation actions could protect as many species as possible. The knowledge of species, however, is incomplete since many species are still unknown (Costello et al. 2013a, b) or poorly known. Myers et al. (2000) claim that biodiversity hotspots, areas characterized by high numbers of endemic species as well as high rates of habitat loss, are prioritary, but a question remains: does the cumulative evaluation of biodiversity, in terms of species additions through time, really represent the expression of biodiversity at a given place?

Appeltans et al. (2012) compiled WoRMS, the World Register of Marine Species (about 226,000 eukaryotic marine species) and used it as a starting point for estimating how many more species may still be discovered. WoRMS published online information on marine species, but many nomenclatural and classification problems remain (Costello et al. 2013a, b). The introduction rate of synonyms is expected to decline through updated taxonomic revisions (Appeltans et al. 2012).

A further problem with the estimation of biodiversity is that local lists are usually updated by adding new entries, but locally (or even finally) extinct species are seldom, if ever, removed from the lists (Boero and Gravili 2013), a task that only taxonomists can undertake through the critical analysis of species lists and the identification of putatively extinct species.

The sea has been far less studied than the land, and our taxonomic knowledge of many groups remains fragmentary (Hilchey 2003). Attempts to inventory all known species led to cover about two-thirds of all marine species (Appeltans et al. 2012), and half of all species (Bisby et al. 2009). Species lists and their distribution are basic to biodiversity research (Costello et al. 2001). May (1994) and Hammond (1994) reviewed a variety of approaches to predict the number of species that may exist on Earth. Moreover, Costello and Wilson (2011) proposed to predict the number of known and unknown species in European seas using rates of description. Biodiversity research has a long history the Mediterranean Sea, one of the best-known seas globally (Coll et al. 2010; Gravili et al. 2013). In particular, the diversity of Mediterranean Hydrozoa is well known and has been recently updated (Bouillon et al. 2004, 2006; Schuchert 2005, 2006, 2008a, b, 2009, 2010; Galea 2007; De Vito et al. 2008; Gravili et al. 2007, 2008, 2010, 2013; Morri et al. 2009; Mastrototaro et al. 2010). The biodiversity of the Mediterranean Sea is high due to ecological, historical, and paleogeographic reasons (Sarà 1985; Bianchi and Morri 2000; Bianchi 2007). The western Mediterranean has strong Atlantic affinities, due to the continued penetration of Atlantic species (Harmelin and d’Hont 1993). Conversely, after the opening of the Suez Canal, the Eastern Mediterranean is receiving species from the Red Sea (Galil 1993). The number of Lessepsian species, now acclimated in the Mediterranean (Golani 1998), is so high that Por (1999) proposed a separate biogeographic province for the Levant Sea.

Many tropical NIS became recently established even in the northwestern Mediterranean waters (Coll et al. 2010; Lejeusne et al. 2010; Zenetos et al. 2012), forming stable populations (Bianchi and Morri 1993) as a response to a warming trend (Sparnocchia et al. 1994; Astraldi et al. 1995).

Ecological and biogeographic theories, supported by significant data, predict that half of all present species may be extinct within the next 100–300 years due to climate change, pollution, over-harvesting, habitat fragmentation and loss (Chapin et al. 2000; Jackson 2008; Costello and Wilson 2011). It is often claimed that extinction rates are on the increase both on land and in the oceans (Carlton et al. 1999; Dulvy et al. 2003; Costello and Wilson 2011), and that chances are good that species might go extinct even before a formal description (Costello et al. 2013a, b). Boero et al. (2013) stressed how well documented marine extinctions usually concern conspicuous species (e.g., the Caribbean monk seal *Monachus tropicalis*, the great auk *Pinguinus impennis*, and the Steller’s sea cow *Hydrodamalis gigas*), and that the number of proven marine extinctions is very low, if compared with the alarming predictions of most review. This is not due to lack of extinction risks but, instead, to poor knowledge of the conservation status of most species (Roberts and Hawkins 1999; Régnier et al. 2009). Boero et al. (2013), however, claimed that the analysis of the history of the records of each species in space and time might be conducive to roughly assess their state of conservation.

Changes in both the abundance and the distribution of species commonly happen due to the arrival of new species, the rarefaction of common species, or the increase in the abundance of formerly rare species (Boero 1994, 1996; Bonsdorff et al. 1997). These changes are a natural feature of all systems but the rate of change can become alarmingly fast (Boero and Bonsdorff 2007). Biotic assessments are increasingly carried out to detect NIS (Gravili et al. 2013; Katsanevakis et al. 2013), and might be used also for the purpose of testing hypotheses of putative extinctions.

The aim of this paper is to review the knowledge of the diversity of Mediterranean Non-Siphonophoran Hydrozoa (NSH), to detect species that are absent since decades, the “missing species”, so as to assess current estimate of the species pool-size and raise cases of either regional or local extinction.

## Methods

The choice of 41 years as a threshold to consider a species as missing was decided based on the rather intense study of hydrozoan species in the Mediterranean in the last four decades, with the establishment of the Hydrozoan Society in 1985 (Boero 2007) that gathered a rather substantial scientific community focusing on the Mediterranean. Due to intensive sampling, thus, if a previously reported species fails to be recorded chances are good that, at least, it is more rare than before. The knowledge about each species is stored in the scientific literature. Every known species has been described in a taxonomic paper, and the date of its first finding is the beginning of the history of its knowledge. The type locality is the centre of origin of that species, even though it might not be representative of the core of its actual distribution. After the original description, species are usually recorded again in other taxonomic, faunistic, or ecological papers. Analyzing the temporal and spatial distribution of species, as recorded by the scientific literature, we can reconstruct maps of their recorded presence in both space and time. We examined current estimates of the size of the Mediterranean species pool, to detect species that might have gone locally or regionally extinct. Picard (1958a) produced the first modern list of Mediterranean NSH. Since then, the number of species almost doubled due to addition of new records to the new ones. To assess the current state of the Mediterranean species pool with the state of the fifties, we compared Picard’s list with the list of the species recorded in the last decade.

**Fig. 1:**
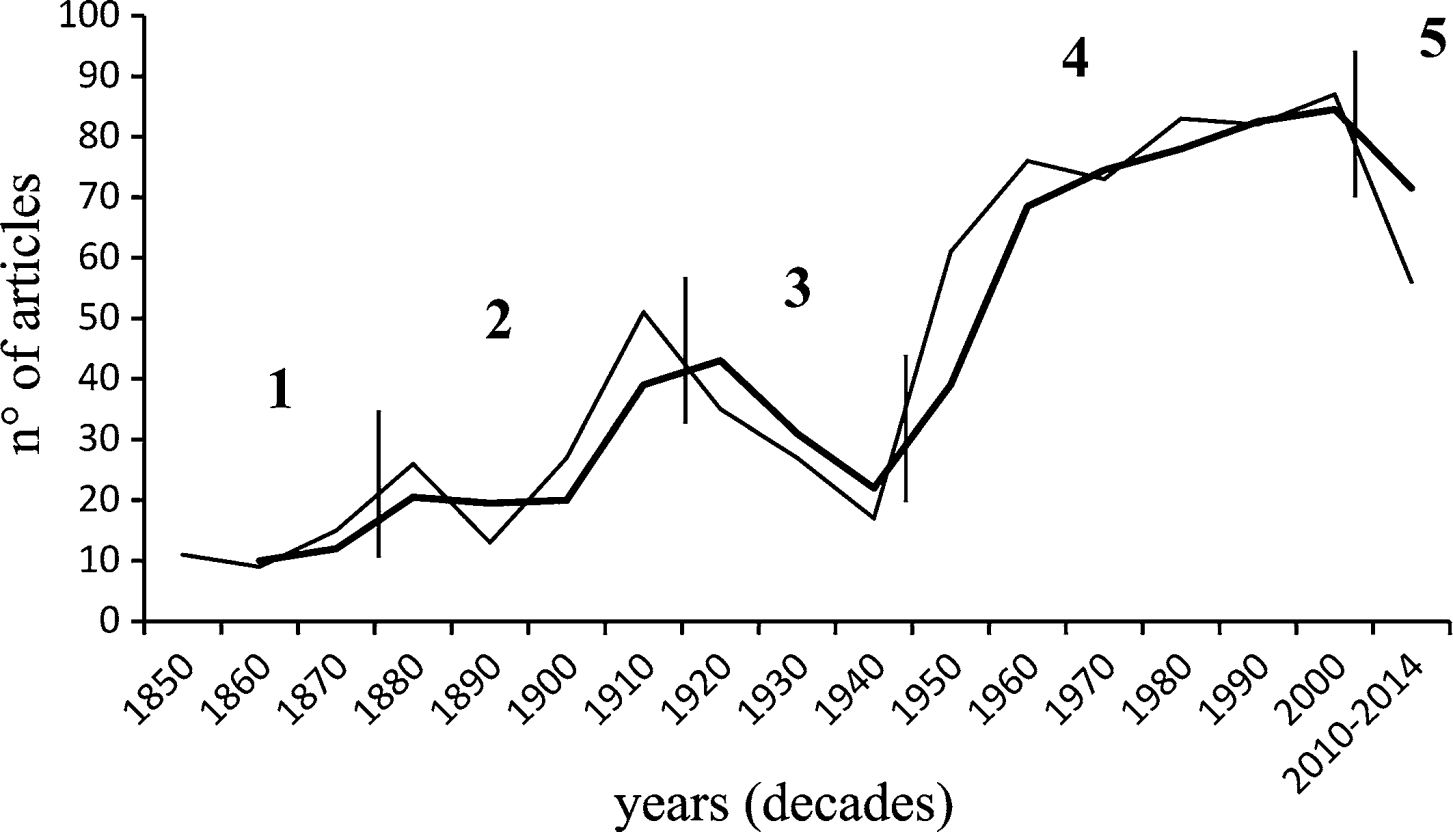
Number of total articles about Mediterranean Hydrozoa since 1850-today by decade: general trend (*thin line*), with mobile average over 2 year periods (*thick line*).* Vertical lines* separate five main periods within the trend

**Fig. 2:**
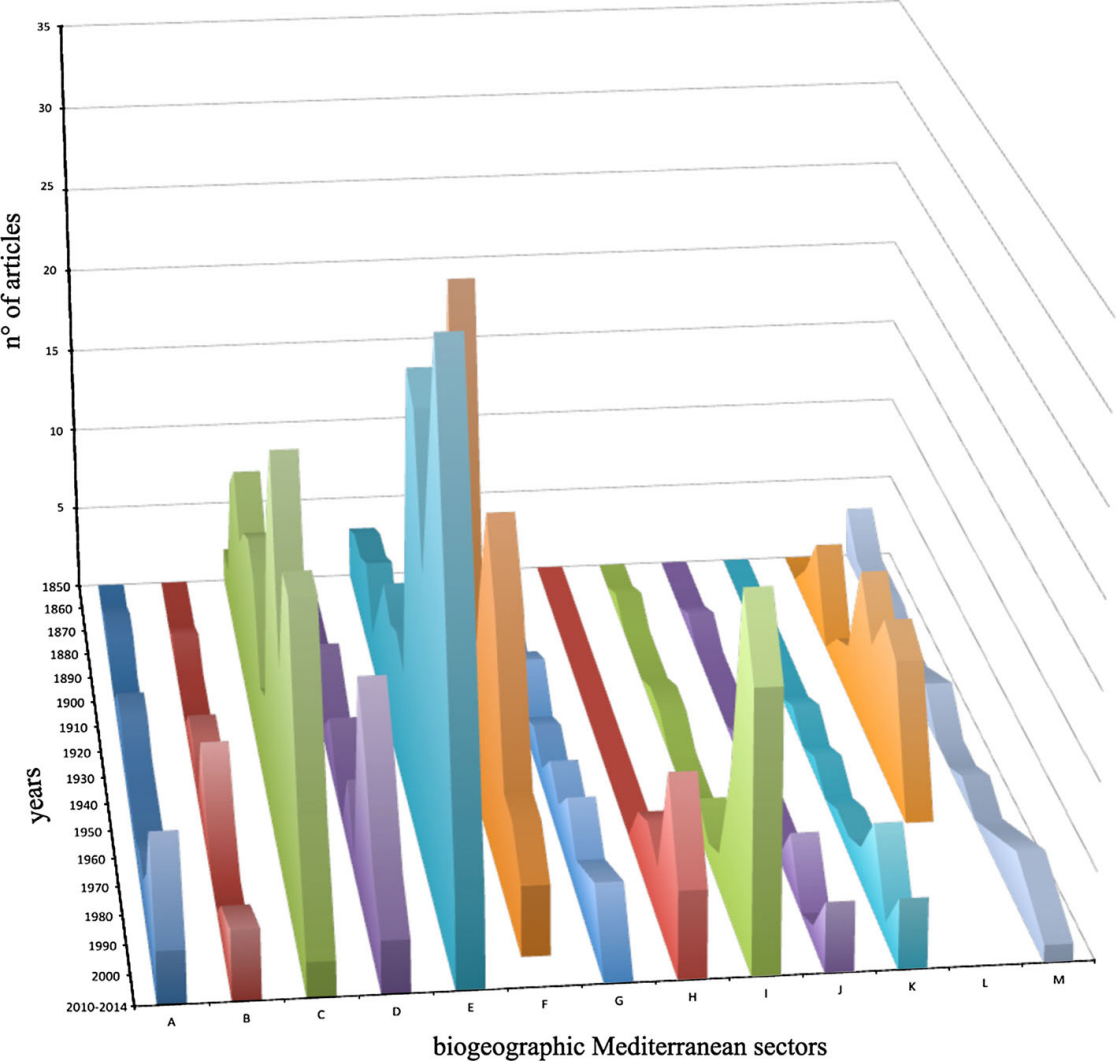
Number of articles that include faunistic studies about Mediterranean Hydrozoa since 1850-today by decade for biogeographic sectors (A–M) according to Bianchi (2007). See map legend (Fig. 3) for abbreviations

Our list of non-Siphonophora Hydrozoan “Missing” species (NSHMs) of the Mediterranean Sea is based on a recent monograph (Bouillon et al. 2004), on taxonomic revisions (e.g. Schuchert 2007, 2008a,b, 2009, 2010), and on an assessment of Mediterranean NSH (Gravili et al. 2013). To determine historical series and distributions, we consulted 749 faunistic studies published between 1850 and 2014. A database with 8,158 records was organized so as to provide the following information: species, family, author, life-cycle phase, reproductive state, location, date of collection e/o year of publication of the article, water depth, substrate type, synonymy, and cited references. Taxonomic records (i.e. records of each taxon, in any kind of report) are reported on a time scale from the original description to the last citation in the literature. The number of faunistic articles on Mediterranean Hydrozoa since 1850 was organized by decade (Fig. 1). The total number of articles (within the same time range) was then referred to each biogeographic sectors (A–M) identified by Bianchi (2007) (Fig. 2).

We identified NSHMs (not recorded since 41 years or more) examining records from the nineteenth century to 2014, to trace the origin, first and last Mediterranean records, current Mediterranean distribution, and global distribution of each species. With few exceptions, we named taxa according to Bouillon et al. (2006). The date and location of the first observation of each NSHMs in the Mediterranean Sea were extracted from the literature. Whenever possible, the actual date of first record was reported, along with its publication date, since the two dates coincide only in a few cases. Strauss and Sadler (1987, 1989) introduced the confidence of extinction index in paleobiology (Marshall 1990), as a method to calculate confidence intervals within local stratigraphic ranges. Boero et al. (2013) adapted this method to analyse cases of putative extinction in recent species. The confidence of extinction index was calculated for each species uncited since 41 years by using the following formula on historical taxonomic data:

$$C \, = \, 1 \, - \, \left( {G/R \, + \, 1} \right)^{ - \, (H - 1)}$$

C is the confidence of extinction, G is the number of years since last sighting, R is the number of years between original description and the last sighting, H is the number of individual years in which there is a record, C ≥ 95 % postulates a case of extinction; 80 % ≤ C ≤ 94 % raises a case of putative extinction.

All records were organized in a presence/absence data matrix of species (NSHMs included) in each biogeographic sector (A–M) for each historical period [>40 years ago (>40 years), from 40 to 31 years ago (40 years), from 30 to 21 years ago (30 years), from 20 to 11 years ago (20 years), and from 10 years ago to nowaday (10 years)]. A distance matrix based on Jaccard’s distance among sector × period centroids was then obtained. A canonical analysis of principal coordinates (CAP) (Anderson and Robinson 2003; Anderson and Willis 2003) based on the distance matrix was then performed for the factor period, in order to portray temporal changes in the whole Mediterranean species pool of NSH. Distinctness among locations was assessed using leave one-out allocation success (Anderson and Robinson 2003). Species most contributing to group differences in the CAP plot were investigated by calculating product–moment correlations (*r*) of original variables (species) with canonical axes (Anderson and Willis 2003). Only species with correlation values exceeding an arbitrarily chosen value of correlation *r* ≥ 0.2 were considered.

The ‘average taxonomic distinctness’ (Δ^+^) (Clarke and Warwick 1998) and ‘variation in taxonomic distinctness’ (Λ^+^) (Clarke and Warwick 2001), complementing Δ^+^, were employed to explore temporal changes in the taxonomic structure of NSH species pool in the whole Mediterranean basin. Δ^+^ represents the average taxonomic path length between two randomly chosen species in the taxonomic tree, whereas Λ^+^ reflects the unevenness in the taxonomic tree of a given species’ list and represents the variance of these pair-wise path lengths. The indices are independent of the number of species in a sample and thus represent useful tools for analysing historical data (Bevilacqua et al. 2009). A reference list, from species to subclass, was made including all NSH species recorded. The list coupled with the presence/absence data matrix was used to calculate the values of Δ^+^ and Λ^+^ of Mediterranean NSH species pool in each period. The same step length (equal to 1) was used in weighting all distances between hierarchical taxonomic levels (Clarke and Warwick 1999). For both taxonomic distinctness indices, the 95 % confidence funnel was generated (Clarke and Warwick 1998, 2001) in order to test temporal departures from expectations of Δ^+^ and Λ^+^ (under the null hypothesis that the species pool in each period was a random subsets of the full NSH species list).

## Results

The updated list of NSH species, after an accurate systematic revision, sums up to 398 species, representing about 11 % of the 3,702 nominal known species of the superclass Hydrozoa reported by Bouillon et al. (2006). The species recorded from the Mediterranean Sea in the last decade sum up to 162, and 118 of them (73 %) are present in Picard’s (1958a) list of 191 species (180 valid species if cleaned up by synonyms); 53 species (13 %) are not recorded in the literature since at least 41 years (Table 1).

**Table 1:** Non-Siphonophoran Hydrozoa Missing species (NSHMs) in the Mediterranean Sea

Taxa	Type locality and original description	Distribution	1st Mediterranean record	Other records in Mediterranean	Confidence of extinction index	Remarks
Class Hydroidomedusae						
Subclass Anthomedusae						
*Bougainvillia multicilia* (Haeckel, 1879)	Algeciras (Gibraltar): 1867 (Haeckel 1879) as *Lizusa multicilia*	Mediterranean-Atlantic	See original description	–	100 %	Doubtful species [see Kramp (1955), Schuchert (2007)]
*Lizzia octostyla* (Haeckel, 1879)	Corfu: 1877 [(Haeckel 1879) as *Dysmorphosa octostyla*]	Endemic of the Mediterranean Sea	See original description	Trieste [Neppi and Stiasny (1911) as *Podocoryne octostyla*; Neppi and Stiasny (1913)]; Villefranche: 1954 Kramp (1957a) as *Koellikerina fasciculata* juv.	78 %	Northern driatic Sea (Benović and Lučić 1996) reported as last record in Adriatic Sea: (Neppi and Stiasny 1913)
*Eudendrium arbuscula* Wright, 1859	Queensferry (close to Edinburgh), Firth of Forth, Scotland: 1858 (Wright 1859)	Boreal (North Atlantic, Mediterranean)	France, Algeria, Syria (Marinopulos 1992) (but gives no records: in the absence of reliable data relating to the records, it has been hypothesized that the records in France, Algeria and Syria had occurred in three individual years)	–	26 %	The Mediterranean records Marinopulos (1992) are likely misidentifications [see Schuchert (2008b)]
*Podocoryna borealis* (Mayer, 1900)	Eastport Harbor, Maine, USA: 1898 (Mayer 1900a) as *Lymnorea borealis*	Boreal (North Atlantic, Mediterranean)	Mediterranean Sea Trégouboff and Rose (1957) as *Podocoryne borealis* but give no records; Naples: 1952 (Riedl 1959) as *P. borealis* (uncertain reports)	–	100 %	The Mediterranean records are unreliable [Schuchert (2008a) as *Hydractinia borealis*]; genus transfer by Schuchert (2013)
*Amphinema turrida* (Mayer, 1900)	Tortugas, Florida, USA: 1897–1899 (Mayer 1900b) as *Dissonema turrida*	Circumtropical (Atlantic. Indo-Pacific, Mediterranean)	Villefranche-sur-Mer: 1964 (Goy 1973)	–	100 %	New Mediterranean material is needed for a further evaluation of the *status* of this form [see Schuchert (2007)]
*Merga galleri* Brinckmann, 1962	Mergellina Harbour, Naples: 1960 (Brinckmann 1962)	Endemic of the Mediterranean Sea	See original description	–	100 %	
*Octotiara russelli* Kramp, 1953	Great Barrier Reef, Southwestern Pacific: 1929 (Australia) (Kramp 1953)	Indo-Pacific, Mediterranean	Bay of Villefranche-sur-Mer: 1954 (Goy 1973) as *Octotiara violacea*	–	100 %	The presence of this species in the Mediterranean is uncertain [see Schuchert (2007)]
*Protiara tetranema* (Péron and Lesueur, 1810)	Coast of The Netherlands: 1809 Péron and Lesueur (1810) as *Oceania Tetranema*	Mediterranean-Atlantic	Adriatic (Pell 1918)	Adriatic: 1913–1914 (Pell 1938)	49 %	Doubtful, unrecognizable species [for more details see Schuchert (2009)]
*Tregoubovia atentaculata* Picard, 1958	Villefranche-sur-Mer: 1955 (Picard 1958b)	Endemic of the Mediterranean Sea	See original description	Villefranche-sur-Mer: 1966 (Goy 1973)	81 %	Very rare species: only two or three specimens have been reported in the literature [see Schuchert (2009)]
*Protohydra leuckarti* Greeff, 1869	Ostende (Greeff 1869)	Boreal (circumglobal in temperate brackish waters of the northern hemisphere), Mediterranean	Canet Plage, Southern France: 1950 (Nyholm 1951)	–	100 %	
*Acauloides ammisatum* Bouillon, 1965	Roscoff, English Channel Bouillon (1965)	Boreal (Northeastern Atlantic, Mediterranean)	Banyuls-sur-Mer: 1961 (Monniot 1962) as ? *Psammocoryne* (invalid nomen nudum).	–	100 %	It is unclear whether *A. ammisatum* occurs in the Mediterranean [for more details see Schuchert (2006)]
*Acauloides ilonae* (Brinckmann-Voss, 1966)	Gulf of Pozzuoli, Naples Brinckmann-Voss (1966) as *Acaulis ilonae*	Endemic of the Mediterranean Sea	Gulf of Pozzuoli, Naples: 1960–1961 (Brinckmann-Voss 1966)	–	100 %	The occurrence of this species outside the Mediterranean is uncertain [for more details see Schuchert (2006)]
*Psammohydra nanna* Schulz, 1950	Western Baltic Sea: 1948 (Schulz 1950)	Boreal (Western Baltic, Northeastern Atlantic, Mediterranean)	Marseille, Western Mediterranean (Swedmark 1956)	Rovigno, Adriatic Sea: 1965 (Salvini-Plawen 1966)	74 %	The taxonomic position of this animal is unclear (Schuchert 2006)
*Eleutheria claparedii* Hartlaub, 1889	Tahitou near St. Vaast la Hogue (Normandy, France) (Hartlaub 1889)	Mediterranean-Atlantic	Naples (Hartlaub 1889)	Naples: Pavesi in a letter to Spagnolini, published 1877 [see Mayer (1910c), Brinckmann-Voss (1970)]	100 %	The polyp has not yet been identified in the sea and only the young polyp without medusae buds is known from cultivation experiments [see Schuchert (2006)]
*Staurocladia portmanni* Brinckmann, 1964	Gulf of Sorrento (polyp stage), Ischia, Naples (medusa stage): 1963 (Brinckmann 1964)	Endemic of Mediterranean Sea	See original description	Gulf of Naples: 1963 Brinckmann-Voss (1987); 1963 [see Bouillon et al. (1995)]	100 %	For more details about its behaviour see Brinckmann-Voss (1970)
*Corymorpha forbesii* (Mayer, 1894)	Nassau Harbour, Bahamas: 1893 (Mayer 1894) as *Hybocodon forbesii*	Circumtropical (Atlantic, Indo-Pacific, Red Sea, Mediterranean)	Gulf of Pozzuoli, Naples: 1962 (Brinckmann-Voss 1967)	–	100 %	For more details about this species see Brinckmann-Voss (1970), Schuchert (2010)
*Branchiocerianthus italicus* Stechow, 1921	Gulf of Naples: 1905 [Lo Bianco (1909) as *Branchiocerianthus* sp.]	Endemic of Mediterranean Sea	See original description	–	100 %	Stechow (1921) introduced the name *B. italicus* for Lo Bianco’s material [for more details see Schuchert (2010)]
*Coryne caespes* Allman, 1871	Gulf of La Spezia (Allman 1871–1872)	Endemic of Mediterranean Sea	See original description	–	100 %	*C. caespes* could belong to *C. pintneri* or *C. muscoides* Schuchert (2001)
*Coryne fucicola* (De Filippi, 1866)	Turin (in an aquarium): 1864 [De Filippi (1866) as *Halobotrys fucicola*]: no type locality specified	Endemic of Mediterranean Sea	See original description	Villefranche-sur-Mer, Balaguir, France (Du Plessis 1888)	84 %	For a complete redescription based on field collected material [see Schuchert (2005)]
*Siphonohydra adriatica* Salvini-Plawen, 1966	Rovigno: 1965 (Salvini-Plawen 1966)	Endemic of Mediterranean Sea	See original description	–	100 %	The gonophores of this animal must be known to assess the validity of the genus and species [see Schuchert (2010)]
*Tricyclusa singularis* (Schultze, 1876)	Bay of Muggia, Trieste: 1875 (Schulze 1876) as *Tiarella singularis*	Boreal (Northeastern Atlantic, Mediterranean)	Bay of Muggia, Trieste: 1875 (Schulze 1876)	–	100 %	After its discovery, it has never been found again in the Mediterranean Sea [for more details see Schuchert (2006)]
*Ectopleura sacculifera* Kramp, 1957	Pacific coast of Ecuador: 1926–1937 (Kramp 1957b)	Indo-Pacific, Mediterranean	Near Naples: 1963 (Brinckmann-Voss 1970)	–	100 %	For more details about this species see Schuchert (2010)
*Tubularia indivisa* Linnaeus, 1758	Northeastern Atlantic (Linnaeus 1758)	Boreal (Northern Atlantic and Pacific, Arctic Sea, Mediterranean)	Cap de Creus, Spanish coast: 1902–1904 Motz-Kossowska (1905)	Naples (Stechow (1923)	35 %	The Mediterranean records need reconfirmation [for more details see Schuchert (2010)]
*Rosalinda incrustans* (Kramp, 1947)	Off Southwestern of Portugal (Kramp 1947)	Atlantic Ocean, West of Gibraltar; Western Mediterranean (Costa Brava and Corsica)	Rosas, Spain: 1958 [see Bouillon et al. (1995)]	West of Corsica (42.355°N 09.611°W): 1958 [see Schuchert (2010)]	100 %	For more details about this species [see Schuchert (2010)]
Subclass Leptomedusae						
*Aequorea pensilis* (Haeckel, 1879)	? Mediterranean Eschscholtz (1829) as *Mesonema pensile*	Indo-Pacific, Mediterranean	See original description	–	100 %	Mediterranean record is doubtful [see Bouillon et al. (2004)]
*Zygocanna vagans* Bigelow, 1912	Philippines Bigelow (1912)	Non classifiable (mainly Indo-Pacific), Mediterranean	Split Canal, Adriatic Sea (Babnik 1948) as *Zygocanna* sp.	–	100 %	
*Calycella syringa* (Linnaeus, 1767)	No type locality specified (Linnaeus 1767) as *Sertularia syringa*	Boreal (occurs North to Arctic Ocean, Mediterranean)	Rovigno [Pieper (1884)—Heller’s collection]	–	100 %	For more details about this species [see Cornelius (1978, 1982, 1995)]
*Eutonina scintillans* (Bigelow, 1909)	Pacific coast of Mexico: 1904–1905 (Bigelow 1909) as *Eutimalphes scintillans*	Indo-Pacific, Mediterranean	Gulf of Trieste: 1910 (Neppi and Stiasny 1911)	Gulf of Trieste: 1910 (Neppi and Stiasny 1913) as *Eutimium scintillans*; Ligurian Sea: 1963 (Goy 1973)	50 %	
*Helgicirrha cari* (Haeckel, 1864)	Nice, France (Haeckel 1864) as *Tima cari*	Mediterranean-Atlantic	See original description	Naples: 1876 [Spagnolini (1877) as *Tima cari*; Mayer (1910c) as *Eirene viridula*]; Tunis: 1923–1924 [Ranson (1925) as *E. viridula*]	90 %	
*Laodicea neptuna* Mayer, 1900	Tortugas, Florida: 1898 (Mayer 1900b)	Mediterranean-Atlantic	Gulf of Naples: 1962 (Brinckmann-Voss 1987)	–	100 %	Doubtful *status* [see Bouillon et al. (2004)]
*Melicertissa adriatica* Neppi, 1915	Adriatic Sea: 1913–1914 (Neppi 1915)	Endemic of Mediterranean Sea	See original description	Adriatic Sea [Neppi (1922) about the Najade Expeditionn results]	100 %	Picard refers this species to *Octogonade mediterranea* [see Addenda in Kramp (1961)]
*Eucheilota maasi* Neppi and Stiasny, 1911	Trieste, Adriatic Sea: 1910 (Neppi and Stiasny 1911)	Endemic of Mediterranean Sea	See original description	Trieste: 1910 (Neppi and Stiasny 1913); Adriatic: 1913–1914 (Pell 1918, 1938)	96 %	
*Eucheilota maculata* Hartlaub, 1894	Heligoland, North Sea Hartlaub (1894) as *Euchilota maculata*	Non classifiable	Illes Medes: 1977–1982 Gili (1982) as *Campanulina*	Illes Medes: 1977–1982 (Gili et al. 1984); North coast of Cape of Creus (Northeastern Spain): 1980–1981 (Gili and Castelló 1985) all as *Campanulina hincksi*	26 %	Hydroid doubtfully reported from Mediterranean; medusa never collected in Mediterranean Sea [see Bouillon et al. (2004)]
*Hydranthea aloysii* (Zoja, 1893)	Naples: 1891 (Zoja 1893) as *Umbrellaria aloysii*	Endemic of Mediterranean Sea	See original description	Trieste [Hadzi (1914) as *Georginella diaphana*]; Marseille: 1953 (Huvé 1954)	74 %	This species is insufficiently described (could be any haleciid or lovenelliid, probably a juvenile of *H. margarica*) [see Bouillon et al. (2004)]
*Orchistomella graeffei* (Neppi and Stiasny, 1911)	Trieste: 1910 (Neppi and Stiasny 1911)	Endemic of Mediterranean Sea	See original description	Ligurian Sea: 1966 (Goy 1973)	46 %	
*Plumularia syriaca* Billard, 1931	Gulf of Alexandrette, Syria coast: 1929 (Billard 1931)	Endemic of Mediterranean Sea	See original description	–	100 %	
*Sertularella tenella* (Alder, 1856)	No type locality was given by Alder (1856), probably Northumberland, England [see Cornelius (1979)]	Cosmopolitan (Northern Atlantic, Caribbean Sea, North Pacific Ocean, Mediterranean)	Monaco: 1929 by Leloup [see Bouillon et al. (1995)]	–	100 %	Doubtful species, probably conspecific with *Sertularella rugosa* [see Cornelius (1995)]
*Thyroscyphus fruticosus* (Esper, 1793)	Type locality unknown (Esper (1793) as *Spongia fruticosa*	Indo-Pacific, Mediterranean	Adriatic: 1885 Marktanner-Turneretscher (1890) as *Campanularia fruticosa*	–	100 %	Schmidt (1973) considered the migration of this species through the Suez-Canal
*Octogonade mediterranea* Zoja, 1896	Messina, Sicily: 1894 (Zoja 1896)	Endemic of Mediterranean Sea	See original description	Dalmatian coast, Adriatic Sea: 1913–1914 (Pell 1918)	83 %	
*Tiaropsidium mediterraneum* (Metschnikoff, 1886)	Messina, Sicily: 1883 (Metschnikoff 1886a) as *Tiaropsis mediterranea*	Endemic of Mediterranean Sea	See original description	Kvarnerola, Adriatic Sea (Hadzi 1916), 1914 as *Camella vilae*-*velebiti* and *Tiaropsis mediterranea*; Gulf of Marseille (Picard 1951b)	73 %	Doubtful record in the South Adriatic Sea, Otranto Channel, Apulia, Italy: 2003 (Piraino et al. 2013)
*Hartlaubella gelatinosa* (Pallas, 1766)	Belgian coast, specimen not located (Pallas 1766) as *Sertularia gelatinosa*	Mainly boreal: Northeastern Atlantic, Western Atlantic and Indo-Pacific (New Zealand), Mediterranean	Lesina Adriatic Sea) [Heller (1868)] as *Laomedea gelatinosa*	Naples (Du Plessis 1881) as *Obelia gelatinosa*; Trieste (Adriatic Sea) (Graeffe 1884) as *Obelia gelatinosa*; Naples: 1905 Lo (Bianco 1909) as *Obelia gelatinosa*; Gulf of Rapallo (Ligurian Sea): 1948 (Rossi 1950) as *Laomedea gelatinosa*	70 %	
*Laomedea neglecta* Alder, 1856	Cullercoats and Tynemouth, UK (Alder 1856)	Boreal (Northeastern Atlantic, Mediterranean)	Rovigno (Adriatic Sea): 1896 (Schneider 1898) as *Campanularia neglecta*	Kotora; Jablanac (Adriatic Sea): 1907 (Babic 1910) as *Campanularia neglecta*; Canale della Corsia, Quarnerolo: 1911 (Broch 1912); Split, Adriatic Sea: 1931 (Broch 1933) as *Laomedea* (*Gonothyrea*) *neglecta*	89 %	
Subclass Limnomedusae						
*Armorhydra janowiczi* Swedmark and Teissier, 1958	Roscoff, France (Swedmark and Teissier 1958)	Mediterranean-Atlantic	Rovigno: 1965 (Salvini Plawen 1966)	Ischia (Clausen 1971)	76 %	
Class Automedusa						
Subclass Actinulidae						
*Halammohydra octopodides* Remane, 1927	Kieler Bucht (Baltic Sea): 1924 (Remane 1927)	Cosmopolitan	Marseille (Swedmark 1956)	Rovigno: 1965 (Salvini Plawen 1966)	54 %	
Subclass Narcomedusae						
*Cunina polygonia* (Haeckel, 1879)	Corfu and Messina: 1877–1878 (Haeckel 1879) as *Cunoctantha polygonia*	Endemic of Mediterranean Sea	See original description	–	100 %	Doubtful *status* [see Bouillon et al. (2004)]
*Cunina proboscidea* (E. & L. Metschnikoff, 1871)	Messina (Gegenbaur 1857) as *Cunina vitrea*	Endemic of Mediterranean Sea	See original description	Mediterranean (E. and L. Metschnikoff 1871); Naples [Mayer (1910c) as *Cunina vitrea* = *C. proboscidea*]; Spanish Mediterranean coast (Ranson 1936); Naples: 1962 [see Bouillon et al. (1995)]	80 %	
*Pegantha rubiginosa* (Kölliker, 1853)	Messina: 1852 Kölliker (1853) as *Eurystoma rubiginosum*	Circumtropical (Atlantic, Indo-Pacific, Mediterranean)	See original description	Messina (Gegenbaur 1857) as *Aegineta prolifera*; Villefranche-sur-Mer, Nice (Haeckel 1864); Naples (Spagnolini 1871), Pavesi (1878) as *Aegineta prolifera*; Naples: 1859 Keferstein and (Ehlers 1861), (Spagnolini 1871) as *Aegineta gemmifera*; (Carus 1884); Capri: 1902 (Lo Bianco 1903) as *Cunina rhododactyla*; Eolie 1902 (Lo Bianco 1903) as *Cunina rhododactyla*; Naples [Lo Bianco (1909) as *Cunina rhododactyla*; Ebbecke (1957) as *C. rhododocatylos*; Vannucci (1966)]; Adriatic Sea (Expedition ‘Najade’) Grobben (1915); Neppi (1915) as *Cunina prolifera*); Villefranche-sur-Mer (Caziot 1921 as *C. prolifera*); Balearic Sea, Tyrrhenian Sea, Strait of Messina, weastern Mediterranean: 1910-1911 (Kramp 1924 as *C. rubiginosa*); Villefranche-sur-Mer: 1954 (Kramp 1957b); Strait of Gibraltar: 1967 (Casanova 1980); Naples: 1956–1962 (see Bouillon et al. 1995); Ligurian Sea: 1963 (Goy 1973)	99 %	
*Pegantha triloba* Haeckel, 1879	Zanzibar, East Africa (Haeckel 1879)	Circumtropical (Atlantic, Indo-Pacific, Mediterranean)	Balearics (Vanhöffen 1913)	–	100 %	For more details about its records [see Kramp (1961)]
*Solmaris corona* (Keferstein and Ehlers, 1861)	Naples: 1859 (Keferstein and Ehlers 1861) as *Aegineta corona*	Circumtropical (Atlantic, Indo-Pacific, Mediterranean)	See original description	Naples (Haeckel 1879 as *Solmaris* *corona* and *S. coronantha*); Balearics: 1909 (Ranson 1936); Strait of Gibraltar: 1967 (Casanova 1980)	66 %	
Subclass Trachymedusae						
*Petasus atavus* Haeckel, 1879	Izmir (Smyrna), Turkey: 1873 (Haeckel 1879) and Canary Islands as *Petasus tetranema*	Mediterranean-Atlantic	See original description	–	100 %	
*Amphogona pusilla* Hartlaub, 1909	Djibuti, East Africa: 1904 (Hartlaub 1909)	Indo-Pacific, Mediterranean	Villefranche-sur-Mer (Ligurian Sea): 1964 (Goy 1973)	–	100 %	
*Arctapodema ampla* (Vanhöffen, 1902)	Bouvet Island (South Atlantic): 1898 (Vanhöffen 1902) as *Homoeonema amplum*	Circumtropical (Antarctic, southern and tropical Atlantic, Mediterranean)	Algeria coast, off Mostaganem: 1908 (Ranson 1936) as *Arctapodema amplum*	Adriatic Sea: 1913-1914 (Pell 1938) as *Isonema najadis* Villefranche-sur-Mer: 1963-1964 (Goy 1971); Nice, Corsica: 1963 (Goy 1973)	69 %	
*Pantachogon militare* (Maas, 1893)	North of Bermudas: 1889 (Maas 1893)	Tropical-Atlantic	Capri: 1902 (Lo Bianco 1903) as *Homoeonema militare*	–	100 %	

*Taxa* Class, subclass, species

*C* Confidence of extinction index (C ≥ 95 % to postulate a case of extinction; 80 % ≤ C ≤ 94 % to raise a case of putative extinction)

? Psammocoryne Monniot, 1962 (invalid nomen nudum). Monniot (1962) identified it as Psammocoryne. This name is not a valid genus as it was not associated with a valid nominal species. Furthermore, Monniot's hydroid could easily also be referred to A. ilonae and it is therefore also somewhat unclear whether A. ammisatum also occurs in the Mediterranean (Schuchert, 2006)

The assessment of the *status* of the unrecorded NSHMs with the Confidence of Extinction Index (C) shows that 32 species (60 %) have C ≥ 95 % so representing cases of extinction; 30 of these have C equal to 100 %; 6 species (11 %) have 80 % ≤ C ≤ 94 % and represent cases of putative extinction; the remaining 15 species (28 %) have C < 80 %. The largest contingent of the missing species is endemic to the Mediterranean (18 species, 34 %), followed by boreal ones (10 species, 19 %), 15 % (8 species) is Mediterranean-Atlantic; the Indo-Pacific and circumtropical contingents are represented by 6 species each (11 %), followed by the cosmopolitan contingent (2 species, 4 %), 1 tropical-Atlantic species (2 %), and 4 % (2 species) are non-classifiable.

Of the 18 endemic NSHMs of Mediterranean Sea, 10 have C ≥ 95 % so representing cases of extinction (*Merga galleri*, *Acauloides ilonae*, *Staurocladia* *portmanni*, *Branchiocerianthus italicus*, *Coryne caespes*, *Siphonohydra adriatica*, *Melicertissa adriatica*, *Eucheilota maasi*, *Plumularia syriaca*, *Cunina polygonia*), the remaining eight ones (*Lizzia octostyla*, *Tregoubovia atentaculata*, *Coryne fucicola*, *Hydranthea aloysii*, *Orchistomella graeffei*, *Octogonade mediterranea*, *Tiaropsidium mediterraneum*, *Cunina proboscidea*), having 80 % ≤ C ≤ 94 %, represent cases of putative extinction (see Table 1).

Moreover, there are difficulties to assess the validity of several NSHMs. It is the case, for example, of *S. adriatica* whose gonophores, as well as the fully-grown animal, remain unknown (for more details see Schuchert 2010). Kramp (1961) reported another case, where Picard (1958a) refers doubtfully *M. adriatica* to the Tiaropsidae as *O. mediterranea* Zoja 1896. *Hydranthea aloysii* is an insufficiently described species that could be any haleciid or lovenelliid (Bouillon et al. 2004). *Bougainvillia multicilia* is considered a doubtful species (see Kramp 1955; Schuchert 2007). Schuchert (2007) retained that new Mediterranean material is needed for a further evaluation of the *status* of the species *Amphinema turrida*. Concerning the species *Protiara tetranema*, recorded by Pell (1918, 1938) from the Adriatic Sea, based on unclear criteria, Schuchert (2009) suggested that it is a doubtful, unrecognizable species. Finally, Bouillon et al. (2004) listed *C. polygonia* as doubtful.

Uncertain records concern species as *Eudendrium arbuscula*, whose Mediterranean records are likely misidentifications (Schuchert 2008b); the records of *Podocoryna borealis* are unreliable according to Schuchert (2008a). The presence of *Octotiara russelli* in the Mediterranean Sea is uncertain. Goy (1973) published the only European record of this species, as *Octotiara violacea*, but this reporting should be re-examined due to the state of preservation of the specimen that impedes certain identification (Schuchert 2007). *Tiaropsidium mediterraneum* was recorded for the first time in Messina (Metschnikoff 1886a) as *Tiaropsis mediterranea*, whereas its record in the South Adriatic (see Piraino et al. 2013) is doubtful.

Moreover, particular problems are related to records of the micro-meiobenthos NSH species that might be underestimated due to paucity of research in this field, namely: *Acauloides ammisatum* (whose presence in Mediterranean Sea is unclear; see Schuchert 2006), *A. ilonae*, *Psammohydra nanna* (whose taxonomic position is unclear; see Schuchert 2006), *Armorhydra janowiczi*, and *Halammohydra octopodides*. A particularly significant example of species that is absent since a very long time is *Tricyclusa singularis* (Schulze 1876). This species of boreal affinity and, since its original description from Trieste, the sole Mediterranean record, it has never been recorded again from the Mediterranean Sea. Its disappearance represents not only a case of Mediterranean extinction of a species, but also of the whole family Tricyclusidae that comprises only this species and genus (Boero and Bonsdorff 2007).

Studies of the Mediterranean Hydrozoa suffered several temporal gaps during the considered period (Fig. 1). The whole trend, expressed in number of papers per decade, can be divided into five periods, marked by changes in the patterns of scientific production (Fig. 1):

- 1850s–1870s, with an average of over 10 papers/decade;
- 1880s–1910s, with an average of about 30 papers/decade: about 20 papers/decade in the sub-period 1880s–1890s, and about 40 papers/decade in 1990s–1910s, with an increase of scientific production until a peak in the 1910s (51 papers) followed by a sharp decrease due to First World War;
- 1920s–1940s, (average of over 25 papers/decade) with a marked decrease coinciding with Second World War;
- 1950s–2000s, with an average of almost 80 papers/decade;
- 2010s–2014s, with an average of about 55 papers/decade, but monitoring of the entire decade (2010–2020) is still incomplete.

Figure 2 shows that since the 19th century many studies were carried out at the Zoological Station of Naples (biogeographic sector C). Messina also attracted high attention (biogeographic sector M), due to the strong currents of its Strait characterized by animals of deep waters. Kölliker (1853), Keferstein and Ehlers (1861), Metschnikoff (1886a, b), worked extensively at Messina contributing to the knowledge of the Hydrozoa. Other Mediterranean places where research on Hydrozoa became prominent were Trieste and Rovinj (biogeographic sector F), Split (biogeographic sector G) and, in France, Villefranche-sur-Mer, Endoume, and Banyuls (biogeographic sector E). In particular, a long series of papers mainly by Picard (1951a,b, 1958b) and Goy (1973) gave a great contribution to the knowledge of the Hydrozoa. Moreover, between the years 1960s and 1970 s several researchers (among these, Bouillon, Brinckmann-Voss, Haeckel, Tardent, Uchida, Vannucci, Yamada) worked at the Naples Zoological Station to describe the life cycles of Hydrozoan species.

**Fig. 3:**
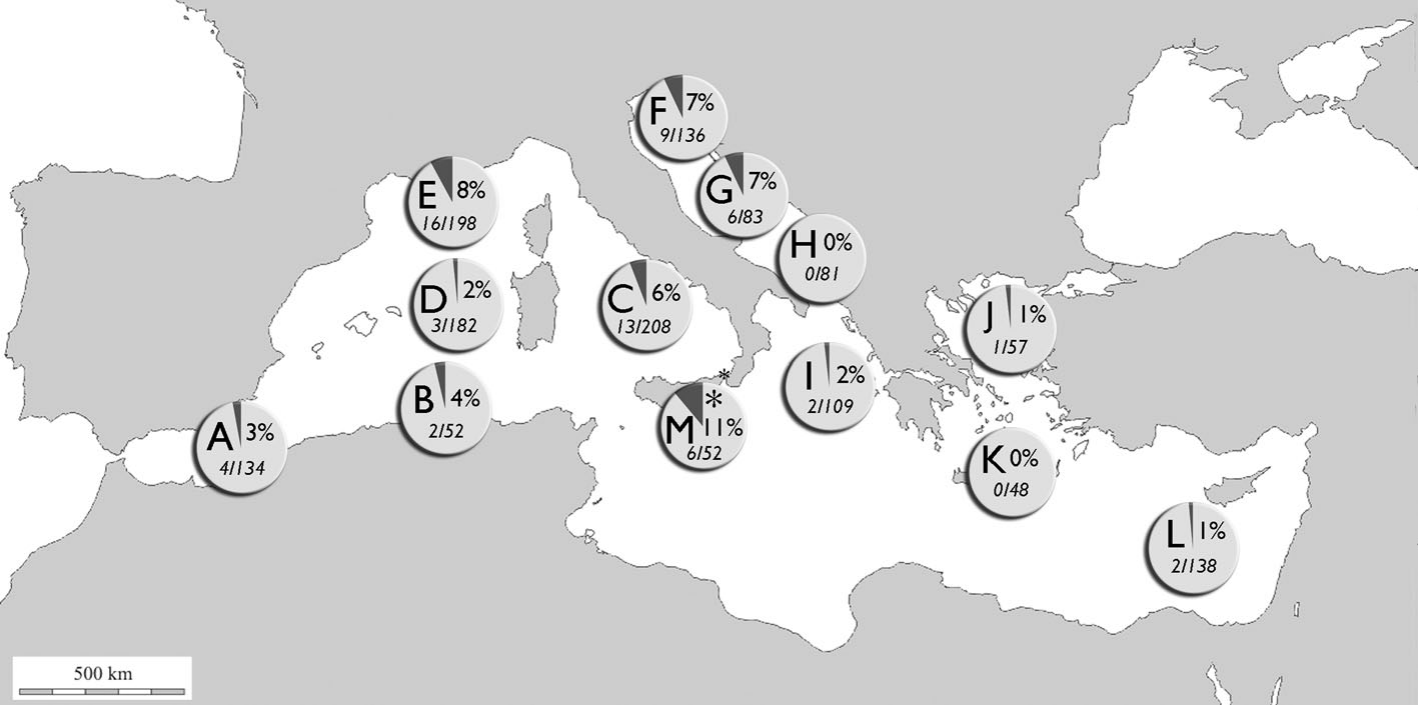
Disappearance of Non-Siphonophoran Hydrozoa Missing species (NSHMs) within the Mediterranean Sea. **a** Alborán Sea; **b** Algeria and North Tunisia coasts; **c** Southern Tyrrhenian Sea; **d** Balearic Sea to Sardinia Sea; **e** Gulf of Lions and Ligurian Sea; **f** Northern Adriatic; **g** Central Adriatic; **h** Southern Adriatic Sea; **i** Ionian Sea; **j** Northen Aegean Sea; **k** Southern Aegean Sea; **l** Levant Sea; **m** Strait of Messina (marked by *asterisk*). Biogeographic sectors according to Bianchi (2007). For each sector, NSHMs percentage and NSHMs number/non-Siphonophoran Hydrozoa (NSH) total number are shown

Numbers of Non-Siphonophoran Hydrozoa Missing species (NSHMs) in each biogeographic sector within the Mediterranean Sea is shown in Fig. 3 reporting percentages of missing species/total number species for each sector. It is clear that the highest percentage of disappearance is linked to the colder biogeographic sectors of the Mediterranean basin: 11 % in the deep waters of the Strait of Messina (biogeographic sector M), 8 % in the Gulf of Lions and Ligurian Sea (biogeographic sector E), 7 % both in the Northern and Central Adriatic Sea (respectively, biogeographic sectors F and G).

**Fig. 4:**
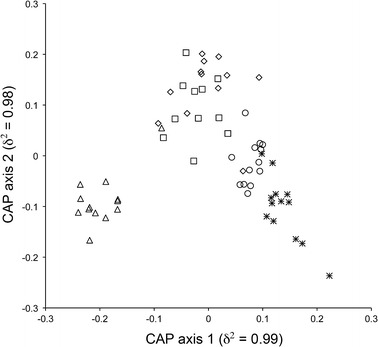
Canonical analysis of principal coordinates (CAP) for the factor period based on the distance matrix (Jaccard’s distance) among sector × period points. *Open triangle* >40 years, *open diamond* 40 years, *open square* 30 years, *open circle* 20 years, *asterisk* 10 years

CAP analysis showed a clear separation of points of > 40 years (left down corner of the plot) from those of 10 years (right down corner), with intermediate position of points of 40, 30, and 20 years (Fig. 4), indicating a temporal gradient of species composition of NSH in the Mediterranean. A total of 171 NSH species showed a correlation value > 0.2 with canonical axes, the 35 % of them being NSHMs (20 %) or NIS (15 %).

**Fig. 5:**
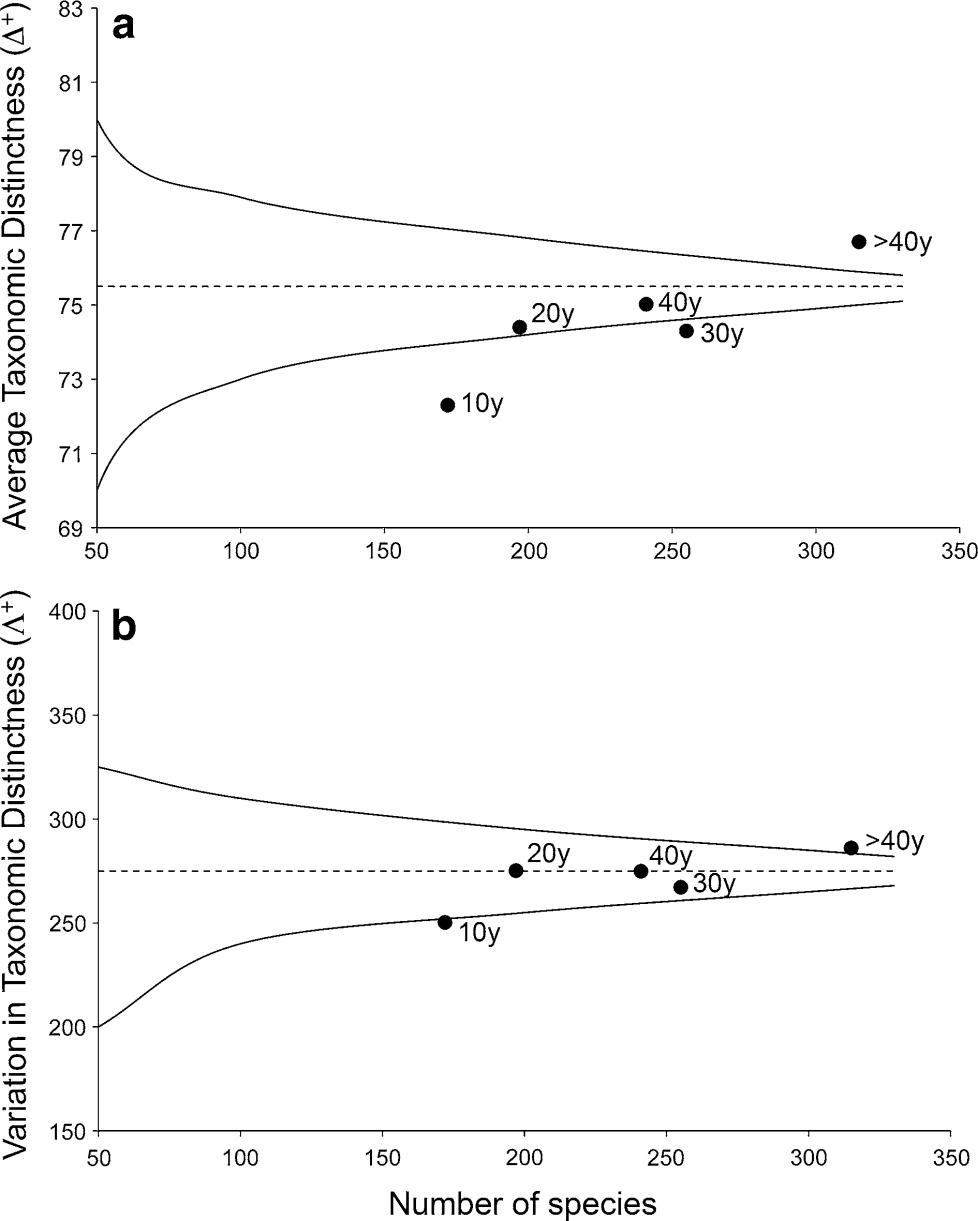
Average taxonomic distinctness (**a**) and variation in taxonomic distinctness (**b**) of Mediterranean NSH species pool in each of the five periods (>40, 40, 30, 20, 10 years) plotted against the corresponding total number of species characterizing each period. For both indices, the expected mean (*dotted line*) and the 95 % confidence limits (*solid lines*) were also plotted from 1,000 independent simulations drawn randomly from the full list of Mediterranean NSH species

Results form analyses on taxonomic distinctness highlighted a decrease of both Δ^+^ and Λ^+^ of the Mediterranean NSH species pool through time (Fig. 5). The species pool of >40y showed significantly higher values (*P* < 0.05) of Δ^+^ and Λ^+^, indicating a higher breadth and heterogeneity of taxonomic structure. In contrast, the species pool in the last decade (10 years) exhibited values of Δ^+^ and Λ^+^ significantly below random expectation, indicating that Mediterranean NSH species were more closely related than expected by chance, with a significant reduction of taxonomic distinctness (Fig. 5).

## Discussion

The scarcity of well-documented cases of extinction in the marine environment shows how difficult it is to deal with the conservation status of marine invertebrates (Boero et al. 2013). Boero and Bonsdorff (2007) wondered if this is the consequence of low global risks of extinction in the sea or, rather, if we fail to notice that species become extinct. According to Roberts and Hawkins (1999), there might have been numerous extinctions in recent times that we failed to realise. Fontaine et al. (2007) addressed the problem of the current indicators that do not cover the species at risk of extinction, as most of rare species are not considered in the European Union’s 2020 target. Alternative indicators about rare species must be developed, in addition to the existing ones that provide information on biodiversity trends (Butchart et al. 2005; De Heer et al. 2005). Indeed, the choice of indicator species should be expanded through a rigorous assessment based on various parameters which take into account also rarity (Fontaine et al. 2007). Moreover, the fundamental question is how soon such changes will occur (Hughes 2000), as well as the particular time ranges chosen for the data sets can greatly affect apparent trends (Hughes 2000). Carlton et al. (1999) observed that the processes of species extinction run at different paces, involving several mechanisms working at different spatial scales. In general, the three main changes in response to environmental stress of the marine communities consist in regression to dominance by opportunist species, reduction of the dominating species resulting in lower diversity (Pearson and Rosemberg 1978; Gray 1989). The features of species that have gone extinct or are nearly extinct (population turnover, reproduction, capacity for recovery, range and distribution, commonness and/or rarity, trophic level) often contribute to their disappearance (Dayton et al. 1995; Roberts and Hawkins 1999).

The Mediterranean Sea is predisposed to local extinction because it is almost closed and much smaller than the open ocean, responding more quickly to environmental change and, furthermore, has a high rate of endemism (Boero and Gravili 2013). This sea is characterized by a particular biota made of highly seasonal species, tropical and boreal contingents being present respectively in the summer, and in the winter (Bavestrello et al. 2006). The Mediterranean marine ecosystem, being subjected to a period of temperature increase that is tropicalizing its biota, represents a model basin for oceans and other seas (Bianchi 2007; Boero and Bonsdorff 2007; Lejeusne et al. 2010).

It is very difficult to confirm the disappearance of a species in the marine environment, mostly due to lack of taxonomist and the existence of synonyms in the species lists (different names attributed to the same species). Therefore, simple lack of suitable sampling or of expertise in recognizing synonyms in previous samplings, might determine their absence from subsequent records. It is debatable whether lack of records is due either to changing of abiotic or biotic factors, or to low sampling efforts or, eventually, to the combination of these causes. Surely, the Mediterranean Sea is going through a radical change that is almost unparalled in respect to any other part of the world (Boero 2014; Templado 2014).

The absence of a species, furthermore, might be only apparent, due to the existence of resting stages that can remain dormant for long periods and that, when activated, are responsible for the so-called “Lazarus effect” (Jablonsky 1986).

As expected by Boero et al. (2008), global warming is favouring the tropical contingent, whereas the boreal one is in distress. If global warming can damage species, the potential sufferers are Mediterranean endemic species (34 % of the NSHMs), those of cold water affinity (19 %) or Mediterranean Atlantic ones (15 %). Indo-Pacific and circumtropical contingents represent each 11 % of the total NSHMs, extinctions in the Mediterranean being probably linked to lack of establishment of species that recently reached the basin. The results of this study confirm the trend characterized at a first time by the abiotic change, induced by increasing temperatures, and followed, at a later time, by biotic change, since the arrival of aliens of tropical affinity (Zenetos et al. 2012; Gravili et al. 2013; Çinar et al. 2014), or the prevalence of the summer contingent that competes against the species of cold water affinity (Puce et al. 2009).

The results of this study, as well as the data analyzed over the long term by Puce et al. (2009), suggest that the regional species pools tend to remain stable in terms of species numbers but not in terms of species identity. In fact, the number of present-day Mediterranean NSH species (162) matches closely the number of species (180 valid species) that Picard (1958a) recorded in his first assessment, cumulating all previous knowledge on the group. However, our finding showed that the composition of the species pool at basin scale changed through time, with changes heavily driven by NSHMs and NIS. Moreover, we detected a progressive contraction of the taxonomic width of NSH, imputable to the loss of taxa poor in species or monotypic, which raises concerns about potential ensuing depletion of taxonomic and functional diversity.

The tropical NIS, colonising the Mediterranean Sea, are probably filling the ecological spaces of species that are becoming rare or are locally extinct. Bianchi (2007) predicted that the northern areas of the Mediterranean Sea will be invaded by warm-water native species, while the southern areas of the basin will be occupied by tropical exotic species. Furthermore, the warming of the Mediterranean Sea might probably cause a decrement of native cold-water species, or even their disappearance (Bianchi 2007).

Boero et al. (2008) proposed the so-called ‘cold engines’ (the northernmost part of the Western Mediterranean, the Northern Adriatic, and the Northern Aegean) as the areas with greater probability of presence of putatively extinct species in a period of global warming. These places, the drivers of the vertical remixing of Mediterranean waters, are significantly colder than the rest of the basin. They are inhabited by many species of cold-water affinity. The compilation of lists of species for all significant taxonomic groups that live only in these areas might provide a tool for creating lists of putatively extinct species, and allow the programming of surveys to ascertain their conservation state (Boero and Gravili 2013). The results shown here suggest that species lists are dynamic, requiring continual updating (introduced species) and putative subtractions of missing species. Without these subtractions, biodiversity is always on the rise due to the arrival of NIS and the species lists will never show possible biodiversity crises at the level of species pools.

Mendelson et al. (2006) required an unprecedented conservation response to stop the loss of species and populations. The rates of marine species description, driven by the increasing ability to explore previously unknown geographic areas, have never been higher, as well as the challenge to estimate the diversity of cryptic species through molecular studies (Appeltans et al. 2012). The rapid influx of NIS and the disappearance of the species of cold-water affinity are heavily influencing the rich but vulnerable Mediterranean ecosystem, heavily affected already by a host of multiple impacts (Claudet and Fraschetti 2010; Boero 2014).

The application of the present analysis to all other taxa will allow for a better assessment of the state of biodiversity in all seas and oceans.
